# Resource Quality and Fungal Richness Are Associated with Soil Organic Matter Dynamics in a Temperate Forest in South Korea

**DOI:** 10.3390/biology15181568

**Published:** 2026-09-08

**Authors:** Ji-Soo Kwak, Jung-Hwa Chun, Ke Dong, Zhi Yu, Lei Chen, Min-Ki Lee, Yong-Ju Lee, Chang-Bae Lee

**Affiliations:** 1 Department of Climate Technology Convergence (Biodiversity and Ecosystem Functioning Major), Kookmin University, Seoul 02707, Republic of Korea; jisoo20211784@kookmin.ac.kr (J.-S.K.); dldydwn0121@kookmin.ac.kr (Y.-J.L.); 2 Forest Ecology Division, National Institute of Forest Science, Seoul 02455, Republic of Korea; chunjh69@korea.kr; 3 Department of Biological Sciences, Kyonggi University, Suwon 16227, Republic of Korea; dongke@kyonggi.ac.kr; 4 Department of Environmental Health Sciences, Graduate School of Public Health, Seoul National University, Seoul 08826, Republic of Korea; zhiyu943@gmail.com; 5 Department of Integrative Biotechnology, Sungkyunkwan University, Suwon 16419, Republic of Korea; chen1213lei@gmail.com; 6 Department of Forest Resources, College of Science and Technology, Kookmin University, Seoul 02707, Republic of Korea; where0203@kookmin.ac.kr

**Keywords:** temperate forest, species richness, functional dispersion, aboveground biomass, fungal ASV richness, soil carbon sequestration, piecewise structural equation modeling

## Abstract

Forest soils play a critical role in carbon sequestration and nutrient cycling by storing large amounts of soil organic matter (SOM). However, the integrated associations linking aboveground resource properties and belowground microbial processes influencing SOM dynamics remain poorly understood. In this observational, cross-sectional study, we analyzed 84 plots across six forest types at Mt. Gariwang, South Korea, to evaluate the relative importance of resource quality and resource quantity in relation to SOM dynamics. Total organic carbon (TOC), total nitrogen (TN), and the C:N ratio were used as indicators of SOM dynamics. Resource quality was represented by species richness (SR) and functional dispersion (FDis) as indirect proxies, whereas resource quantity was represented by aboveground biomass (AGB) and diameter at breast height diversity (DBH diversity). We also evaluated whether fungal and bacterial ASV richness were statistically linked to the relationships between aboveground resource properties and SOM dynamics. Functional dispersion (FDis) was negatively associated with TOC (β = −0.28) and the C:N ratio (β = −0.51), whereas AGB showed no significant relationship with any SOM indicator. In contrast, SR was positively associated with Fungal ASV richness (β = 0.37), which was in turn positively linked to TOC (β = 0.43) and TN (β = 0.56) accumulation, suggesting an indirect statistical pathway consistent with fungi acting as a biological link between aboveground biodiversity and belowground carbon and nitrogen storage. These findings indicate that resource quality indicators showed divergent associations with SOM dynamics: a negative direct pathway via FDis and a positive indirect pathway via SR and fungal ASV richness, and that, overall, resource quality was more consistently associated with SOM dynamics than resource quantity. Our study provides new insights into the associations linking aboveground vegetation and belowground microbial communities and offers a scientific basis for biodiversity-based forest management to enhance long-term soil carbon sequestration.

## Simple Summary

Forest soils store large amounts of carbon and play an important role in regulating climate and maintaining healthy forest ecosystems. Understanding what controls the accumulation of soil organic matter is therefore essential for improving forest management and supporting climate change mitigation. In this study, we investigated whether soil organic matter is influenced more by the resource quality or by the resource quantity in a temperate mountain forest in South Korea. We also examined how soil microorganisms, including fungi and bacteria, contribute to this process. We found that the resource quality was more strongly associated with soil organic matter than the resource quantity. Forests with greater resource quality supported richer fungal communities, which were associated with greater accumulation of carbon and nitrogen in the soil. These findings suggest that maintaining diverse forests with a wide range of plant characteristics is more effective for improving long-term soil health and carbon storage than simply increasing forest biomass. A better understanding of these relationships can help develop more sustainable forest management strategies, strengthen biodiversity conservation, and improve the ability of forests to store carbon under changing environmental conditions.

## 1. Introduction

Forest ecosystems harbor approximately 80% of terrestrial biodiversity and serve as a major carbon sink, absorbing approximately one-third of global greenhouse gas emissions [1]. Forest soils constitute one of the largest terrestrial carbon reservoirs, highlighting the importance of understanding soil carbon sequestration mechanisms. A significant portion of global forest carbon is stored in living biomass 43% and soil 45% [2]. In particular, soil functions as a major reservoir that simultaneously stores both carbon and nitrogen [3]. Within this soil matrix, soil organic matter (SOM), the primary form in which carbon and nitrogen are retained, links global carbon and nitrogen cycles [4]. Beyond nutrient storage, SOM regulates soil fertility and ecosystem productivity through organic matter accumulation, decomposition, and nutrient cycling, thereby contributing to the long-term functioning and stability of forest ecosystems [5]. Consequently, understanding the factors regulating SOM dynamics is essential for predicting ecosystem responses to environmental change and developing effective forest management and climate change mitigation strategies [6]. Although a wide array of physical, chemical, and biological parameters has been employed to evaluate SOM dynamics, total soil organic carbon (TOC), total nitrogen (TN), and the C:N ratio remain among the most widely used chemical indicators for assessing soil carbon storage, nutrient cycling, and ecosystem functioning [7]. TOC represents soil organic carbon accumulation and indicates soil carbon storage and sequestration potential [8]. TN reflects the soil nitrogen reservoir and is closely associated with organic matter content and nutrient availability [9]. The C:N ratio characterizes the stoichiometric relationship between carbon and nitrogen in SOM and reflects organic matter decomposition and nutrient cycling [10].

However, SOM dynamics are influenced not only by soil physicochemical properties but also by the characteristics of organic matter inputs derived from aboveground vegetation [11]. Recently, research on biodiversity–ecosystem functioning (BEF) has highlighted tree diversity as a key factor influencing SOM dynamics, including soil carbon storage and nutrient cycling [12]. Tree diversity, characterized by traits such as species, functional, and structural diversity, alters SOM dynamics by modulating the quality and quantity of organic matter entering the soil [13,14]. Specifically, structural diversity and tree biomass can increase the quantity of organic inputs to the soil by enhancing litter production and root biomass [15,16]. Although species richness and functional dispersion do not directly measure the chemical composition of organic inputs, they are widely used as indirect proxies of resource quality, as greater species and functional diversity are expected to diversify litter traits and decomposition pathways [17]. Consequently, biodiversity is considered a crucial ecological mechanism that simultaneously regulates both the quantity and quality of organic resource entering the soil [18].

The mechanism by which biodiversity drives SOM dynamics can be primarily explained through two conceptual frameworks: niche complementarity and the mass ratio hypothesis [19,20,21,22]. Directly testing the mass ratio hypothesis typically requires community-weighted trait means or the identification of dominant species’ traits; such analyses were beyond the scope of the present study. Accordingly, higher aboveground species and functional diversity can alter the quality of organic matter inputs and improve resource-use efficiency, consequently promoting SOM formation [23]. From this perspective, communities characterized by higher aboveground biomass (AGB) and structural diversity are expected to contribute to SOM accumulation by supplying a greater volume of organic matter to the soil [24]. Therefore, SOM dynamics can be influenced not only by resource quality, which is associated with functional and species diversity, but also by resource quantity, which is determined by biomass and stand structure.

Meanwhile, the effects of tree diversity on SOM dynamics are often linked to soil microbial communities [25]. Soil fungi and bacteria are recognized as key organisms regulating organic matter decomposition, nutrient mineralization, and microbial carbon stabilization [26,27]. Consequently, vegetation characteristics can alter the composition and activity of soil microbial communities, thereby indirectly influencing SOM accumulation and nutrient cycling processes [28,29].

However, most studies to date have focused primarily on either resource quantity or resource quality in isolation rather than evaluating their relative contributions concurrently. For example, tree species evenness enhanced organic horizon soil carbon and nitrogen by up to 30% and 42% on a decadal scale [12], whereas in another study, aboveground biomass and structural diversity, rather than species diversity, were identified as the primary drivers of topsoil carbon accumulation [16]. Research simultaneously evaluating the relative importance of these two factors within a single system, however, remains limited. Furthermore, there is still a lack of studies that integratively consider the mediating role of soil microbiota in the relationship between vegetation characteristics and SOM. In this context, this study aims to compare the relative contributions of resource quality (functional and species diversity) and resource quantity (AGB and structural diversity) to SOM dynamics (TOC, TN, and the C:N ratio) in Mt. Gariwang, a temperate mountain forest in South Korea, while elucidating the mediating role of soil microbiota (fungi and bacteria ASV richness) in this process. We test two primary hypotheses: (i) resource quality (SR and FDis) and resource quantity (AGB and DBH diversity) are expected to show positive associations with TOC and TN, and a negative association with the C:N ratio; (ii) these associations are expected to be more strongly linked to fungal ASV richness than to bacterial ASV richness.

## 2. Materials and Methods

### 2.1. Study Area, Data Collection, and Calculation of SOM Dynamics

**Figure 1:**
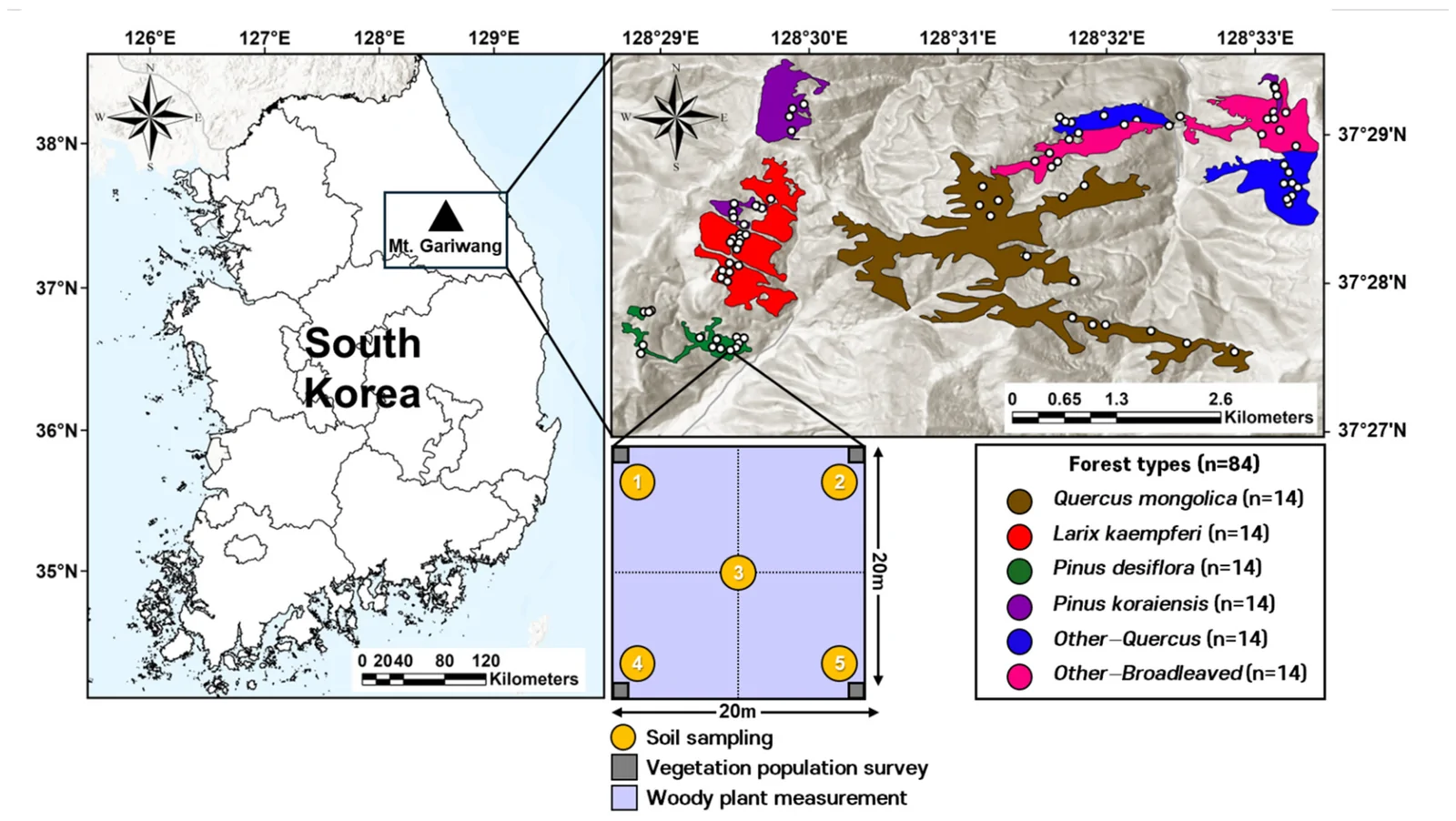
Locations of 84 study plots established across six forest types in Mt. Gariwang, South Korea: Other—*Quercus*, Other—*broadleaved*, *Quercus mongolica*, *Pinus densiflora*, *Larix kaempferi*, and *Pinus koraiensis* (*n* = 14 for forest type). White dots indicate the locations of the individual study plots. In each 20 × 20 m^2^ plot, woody plants were surveyed within the main plot shown in purple, herbaceous plants were surveyed in four 1 × 1 m^2^ quadrats shown in gray, and soil samples were collected from five points indicated by yellow circles.

The study area was Mt. Gariwang, located in Jeongseon-eup, Pyeongchang-gun, South Korea (37°27.5′–37°30.5′ N, 128°30.5′–128°33.5′ E, Figure 1), and is a representative natural forest in the temperate central and northern climate zones of South Korea. The study area is underlain by Triassic Pyeongan Group bedrock, predominantly composed of sandstone and shale. The elevation ranges from 500 m to 1561 m, forming a diverse environmental gradient that includes the subalpine forest zone [30]. The soil in this mountainous region is predominantly derived from Paleozoic sedimentary bedrock and is classified as Cambisols and Umbrisols according to the World Reference Base for Soil Resources (WRB) classification system [31]. Field surveys, including both vegetation measurements and soil sampling, were conducted during the growing season each year from June 2021 to 2022. The vegetation types vary with elevation, ranging from temperate deciduous and coniferous forests to subalpine zones, where several rare plant species with high conservation value also grow naturally [32].

Data collection was conducted across a total of 84 plots, with 14 plots established in each of six forest types (Other—*Quercus*, Other—*broadleaved*, *Quercus mongolica*, *Pinus densiflora*, *Larix kaempferi*, and *Pinus koraiensis*). Vegetation surveys and soil sampling were conducted within 20 m × 20 m plots. In each plot, all woody plants with a diameter at breast height (DBH) greater than 2 cm were identified to species level and counted. Four 1 m × 1 m quadrats were established at the corners of each plot, giving a total sampled area of 4 m^2^ for herbaceous plants, and the number of individuals of each herbaceous plant species was recorded [33]. For soil analysis, samples were collected from five points in each plot, including the center and four corners, and then pooled to form a single composite sample per plot. Previous studies have reported that the soils in Mt. Gariwang are predominantly silty loam regardless of slope position or soil horizon, reflecting the sedimentary parent materials of the region [34]. Prior to sampling, the surface litter layer was removed, and soil samples were then collected from the 0–30 cm mineral soil layer, where root systems and nutrient availability are most prominent [35,36].

To characterize soil organic matter (SOM) dynamics, we evaluated total organic carbon (TOC), total nitrogen (TN), and the carbon-to-nitrogen ratio (C:N ratio; calculated as the ratio of TOC to TN) as key indicators. TOC was quantified using the dry combustion method at the National Instrumentation Center for Environmental Management (NICEM), Seoul National University, while TN was measured using the Dumas combustion method at the Korea Forestry Promotion Institute. Subsequently, the C:N ratio was calculated based on the measured TOC and TN values to assess the characteristics of soil organic matter dynamics.

### 2.2. Quantification of Resource Quality and Quantity

To examine the drivers of SOM dynamics, this study focused on two major ecological components: resource quality and quantity. Resource quality was represented by functional dispersion (FDis) and species richness (SR) as indirect proxies of functional and species diversity, respectively, because greater functional and species diversity are expected to diversify the chemical composition and decomposability of litter and root inputs entering the soil [14]. In contrast, resource quantity was represented by aboveground biomass (AGB) and diameter at breast height diversity (DBH DIV), the latter reflecting stand structural diversity, as both are expected to primarily determine the total volume of organic matter (e.g., litter and root biomass) supplied to the soil [13]. We note that SR, FDis, AGB, and DBH DIV are indicator variables rather than direct measurements of litter chemical composition or organic matter flux. This analysis aimed to determine whether SOM dynamics were more strongly influenced by resource quality or resource quantity.

FDis was calculated following the methods of previous studies [37]. Specifically, six FDis indices were calculated, including one multi-trait FDis based on five functional traits, maximum height (MH), specific leaf area (SLA), wood density (WD), leaf carbon content (C), and leaf nitrogen content (N), as well as five single-trait FDis indices calculated separately for each trait. Except for MH, leaf and wood samples for the remaining four functional traits were collected in our laboratory. Their physicochemical properties, including WD, C, and N, were analyzed by the Korea Forestry Promotion Institute, an authorized analytical agency, following globally standardized protocols [38]. AGB was estimated by measuring all trees with DBH ≥ 2 cm within each plot and applying species-specific allometric equations derived from previous studies (Table S1). DBH DIV was calculated using the Shannon–Wiener index based on 2 cm DBH class intervals.

### 2.3. Soil Microbiota Metabarcoding and ASV Richness

To evaluate soil microbial communities as potential statistical links between resource properties and SOM dynamics, DNA was extracted from 0.25 g of each homogenized composite soil sample using the Maxwell^®^ RSC PureFood GMO and Authentication Kit (Promega, Fitchburg, WI, USA) following the manufacturer’s protocol. Extracted DNA was stored at −80 °C until PCR amplification. For PCR and library preparation, a no-template control consisting of sterile distilled water and a positive control consisting of control DNA provided by Macrogen, Inc. (Seoul, Republic of Korea) were included. For fungi, the ITS2 region was amplified using ITS3 (5′-GCATCGATGAAGAACGCAGC-3′) and ITS4 (5′-TCCTCCGCTTATTGATATGC-3′), whereas the V3–V4 region of the bacterial 16S rRNA gene was amplified using 341F (5′-CCTACGGGNGGCWGCAG-3′) and 805R (5′-GACTACHVGGGTATCTAATCC-3′). Complete fusion-primer structures, including Illumina adapters and index sequences, are provided in Table S2. PCR amplification consisted of an initial denaturation at 95 °C for 3 min, followed by 25 cycles of denaturation at 95 °C for 30 s, annealing at 55 °C for 30 s, and extension at 72 °C for 30 s, with a final extension at 72 °C for 5 min. PCR products were verified using 1% agarose gel electrophoresis, purified using CleanPCR (CleanNA, Waddinxveen, The Netherlands), pooled at equimolar concentrations, and assessed using a DNA 7500 chip on a Bioanalyzer 2100 (Agilent, Palo Alto, CA, USA). The pooled amplicon libraries were sequenced using the Illumina MiSeq platform by Macrogen, Inc. (Seoul, Republic of Korea).

Raw paired-end reads were processed using Cutadapt v3.2 and DADA2 v1.18.0. Adapter and primer sequences were removed using Cutadapt, followed by quality filtering, error-model-based denoising, paired-end merging, and chimera removal using DADA2. Reads with expected errors > 2 were discarded, and chimeric sequences were removed using the consensus method implemented in the removeBimeraDenovo function of DADA2. For the bacterial dataset, ASVs shorter than 350 bp were additionally removed using R v4.0.3, whereas no corresponding length-based filtering was applied to the fungal ITS ASVs. No additional abundance- or prevalence-based filtering of rare ASVs was applied after these quality-control procedures. Fungal ASVs were taxonomically assigned using UCLUST against the UNITE Fungi2 99% Clustering database (v8.2, 20 February 2023); an additional taxonomic assignment against the UNITE + INSD All Eukaryotes database (v8.2, 4 February 2023) was also generated as part of the fungal sequencing pipeline. Bacterial ASVs were assigned using BLAST + v2.9.0 (National Center for Biotechnology Information, Bethesda, MD, USA) against the NCBI 16S reference database (NCBI_16S_20220526; release 26 May 2022). Additional details of sequence processing are provided in the Supplementary Methods (Supplementary Material S2).

Sequencing depth was standardized within each sequencing batch by rarefaction to the minimum retained read count using QIIME v1.9.0 [36]. Across the 84 study plots, approximately 7.2 × 10^4^–1.57 × 10^5^ raw fungal reads and 5.9 × 10^4^–1.53 × 10^5^ raw bacterial reads were obtained per sample. After quality filtering, denoising, paired-end merging, and chimera removal, approximately 4.5 × 10^4^–1.21 × 10^5^ fungal reads and 1.7 × 10^4^–6.2 × 10^4^ bacterial reads per sample were retained [39,40,41].

Fungal and bacterial diversity was quantified from rarefied ASV tables as the number of ASVs detected in each plot, hereafter referred to as fungal ASV richness and bacterial ASV richness, respectively. These sequence-derived richness metrics quantify detected ASV richness and should not be interpreted as direct measures of microbial abundance, biomass, or metabolic activity.

Summary statistics for resource quality, resource quantity, soil microbial ASV richness, and SOM indicators are presented in Figure S1 and Table S3.

### 2.4. Statistical Analysis

Prior to statistical analysis, all variables used in the study were log- or square root-transformed to improve linearity and normality. To ensure consistency, all variables were subsequently standardized [42]. To evaluate the presence of spatial autocorrelation, generalized least squares (GLS) was applied by comparing model formulations with and without spatial variables (i.e., the geographic coordinates of each plot). Model performance, assessed using the Akaike Information Criterion (AIC), indicated that incorporating spatial structure did not substantially enhance the explanatory power of the model (Table S4). Pearson correlation analysis was conducted to identify strong correlations among variables (Figure S2). To evaluate multicollinearity in the multiple regression models, variance inflation factors (VIFs) were calculated for each predictor variable, and all values were below 3, indicating that multicollinearity was not a major concern [43,44].

Multi-model inference was conducted to evaluate the relative importance of predefined predictors representing resource quality (FDis and SR), resource quantity (AGB and DBH diversity), and soil microbiota (fungal and bacterial ASV richness) in explaining soil organic matter variables TOC, TN, and the C:N ratio.

**Figure 2:**
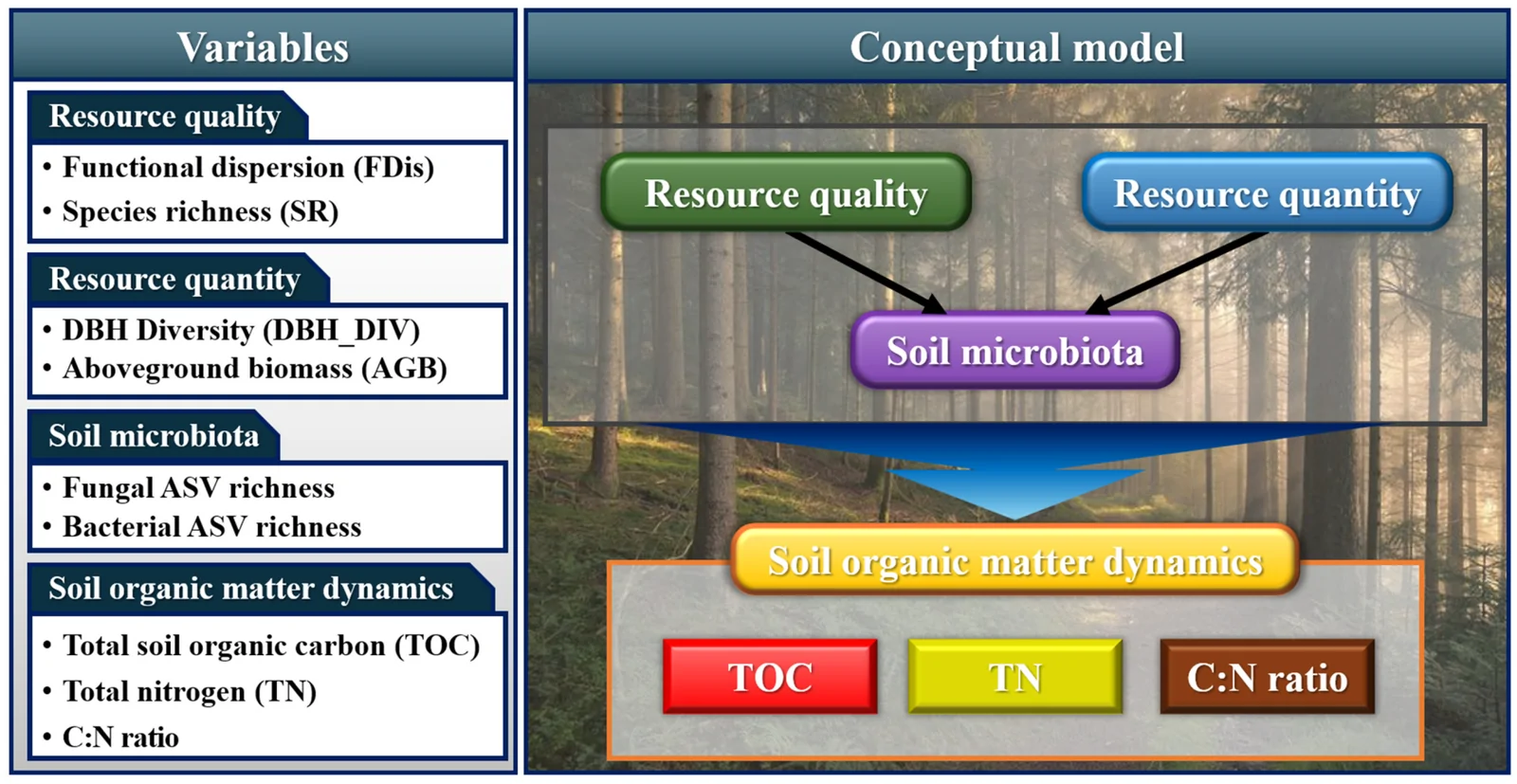
Conceptual model indicating how multiple resource quality, resource quantity, and soil microbiota affect TOC, TN, and the C:N ratio in Mt. Gariwang, South Korea. Abbreviations: FDis, functional dispersion; SR, species richness; DBH DIV, the diversity of diameter at breast height; AGB, aboveground biomass; TOC, total soil organic carbon; TN, total nitrogen; C:N ratio, total organic carbon-to-total nitrogen ratio.

Based on the conceptual model (Figure 2), piecewise structural equation modeling (pSEM) was used to evaluate the direct and indirect relationships among these variables. The initial pSEM included all hypothesized pathways. Non-significant paths were sequentially removed, following the standard model-simplification procedure widely applied in forest ecological pSEM studies [45,46,47,48], and competing models were compared using Akaike’s Information Criterion (AIC), Fisher’s C statistic, and associated *p*-values [44]. The final model was selected based on the lowest AIC together with acceptable Fisher’s C statistics and non-significant *p*-values, indicating a good fit to the observed data.

Multi-model inference and pSEM were implemented using the “MuMIn” (Burnham and Anderson, 2004) and “piecewiseSEM” packages [46], respectively, in R version 4.3.3 [47]. For both the multi-model inference and pSEM analyses, linear mixed-effects models (LME) were fitted, with forest type (six forest types) included as a random effect. Spatial autocorrelation structures were not incorporated into the final models, as comparison of spatial and non-spatial GLS models indicated no substantial improvement in model fit when geographic coordinates were included (Table S4). Model performance was evaluated using marginal R^2^ (R^2^_m_) and conditional R^2^ (R^2^_c_), representing the variance explained by the fixed effects alone and by the combined fixed and random effects, respectively [45,48].

## 3. Results

**Figure 3:**
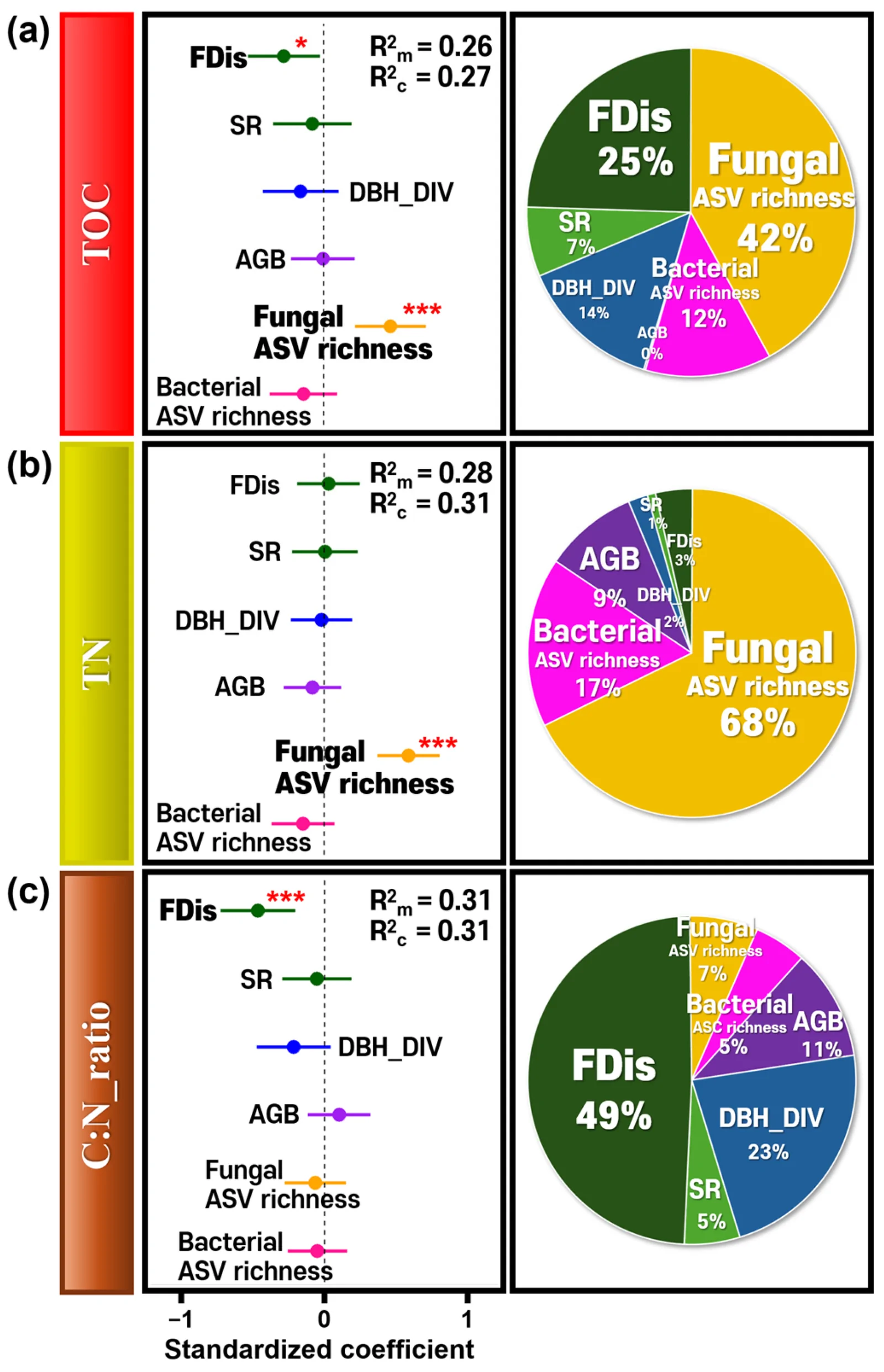
Model−averaged parameter estimates and relative importance of predictors affecting (**a**) total organic carbon (TOC), (**b**) total nitrogen (TN), and (**c**) the C:N ratio. Circles indicate standardized effect sizes, horizontal bars represent 95% confidence intervals, and pie charts show the relative importance of each predictor. Abbreviations are provided in Figure 1. Significance levels: * *p* < 0.05 and *** *p* < 0.001.

Multi-model inference identified fungal ASV richness and FDis as the primary predictors of SOM variables (Figure 3; full model estimates are provided in Table S5). Fungal ASV richness was the most important predictor of both TOC and TN and showed a significant positive effect (Figure 3a,b), whereas FDis was the most important predictor of the C:N ratio and exerted a significant negative effect (Figure 3a,c).

**Figure 4:**
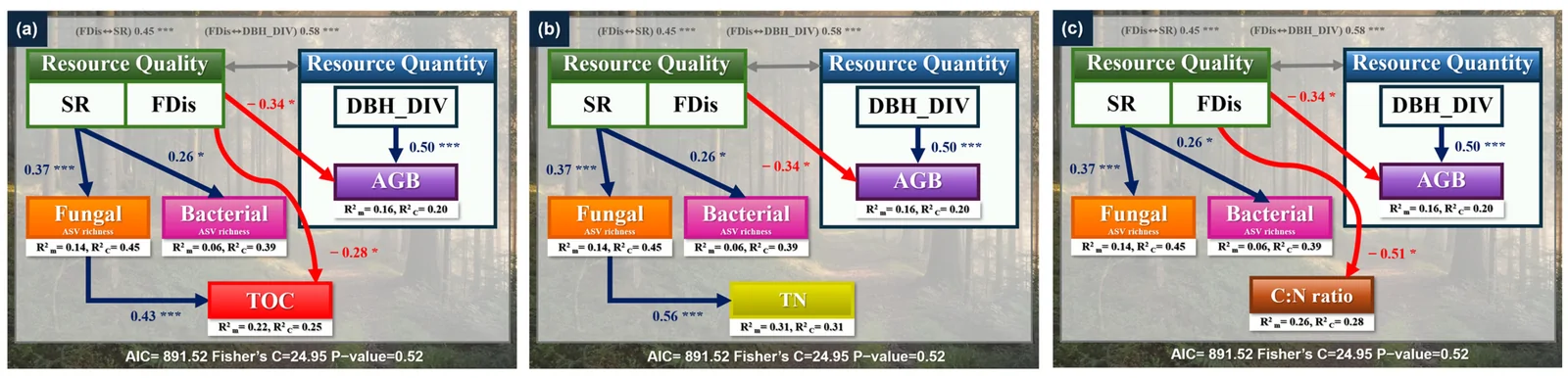
Piecewise structural equation models accounting for the effects of resource quality, resource quantity, and soil microbial communities on (**a**) total organic carbon (TOC), (**b**) total nitrogen (TN), and (**c**) the C:N ratio. Blue and red arrows indicate positive and negative relationships, respectively, and solid arrows represent significant paths (*p* < 0.05). Standardized coefficients are shown for each arrow and covariance. Abbreviations: AIC, Akaike information criterion; Fisher’s C, Fisher’s chi-square. Other abbreviations are defined in Figure 1. Significance levels: * *p* < 0.05 and *** *p* < 0.001.

Overall, the pSEM revealed that soil organic matter (SOM) dynamics were driven more consistently by resource quality and soil microbiota than by resource quantity. A common baseline relationship was observed across all three models, with DBH diversity positively affecting AGB (β = 0.50, *p* < 0.001), whereas FDis negatively affected AGB (β = −0.34, *p* < 0.05), and SR positively influenced both fungal ASV richness (β = 0.37, *p* < 0.001) and bacterial ASV richness (β = 0.26, *p* < 0.05; Figure 4). For TOC, FDis had a significant negative direct effect (β = −0.28, *p* < 0.05), whereas fungi had a positive effect (β = 0.43, *p* < 0.001; Figure 4a). Similarly, TN was positively affected by fungal ASV richness (β = 0.56, *p* < 0.001; Figure 4b). For the C:N ratio, FDis exerted a significant negative effect (β = −0.51, *p* < 0.001).

**Figure 5:**
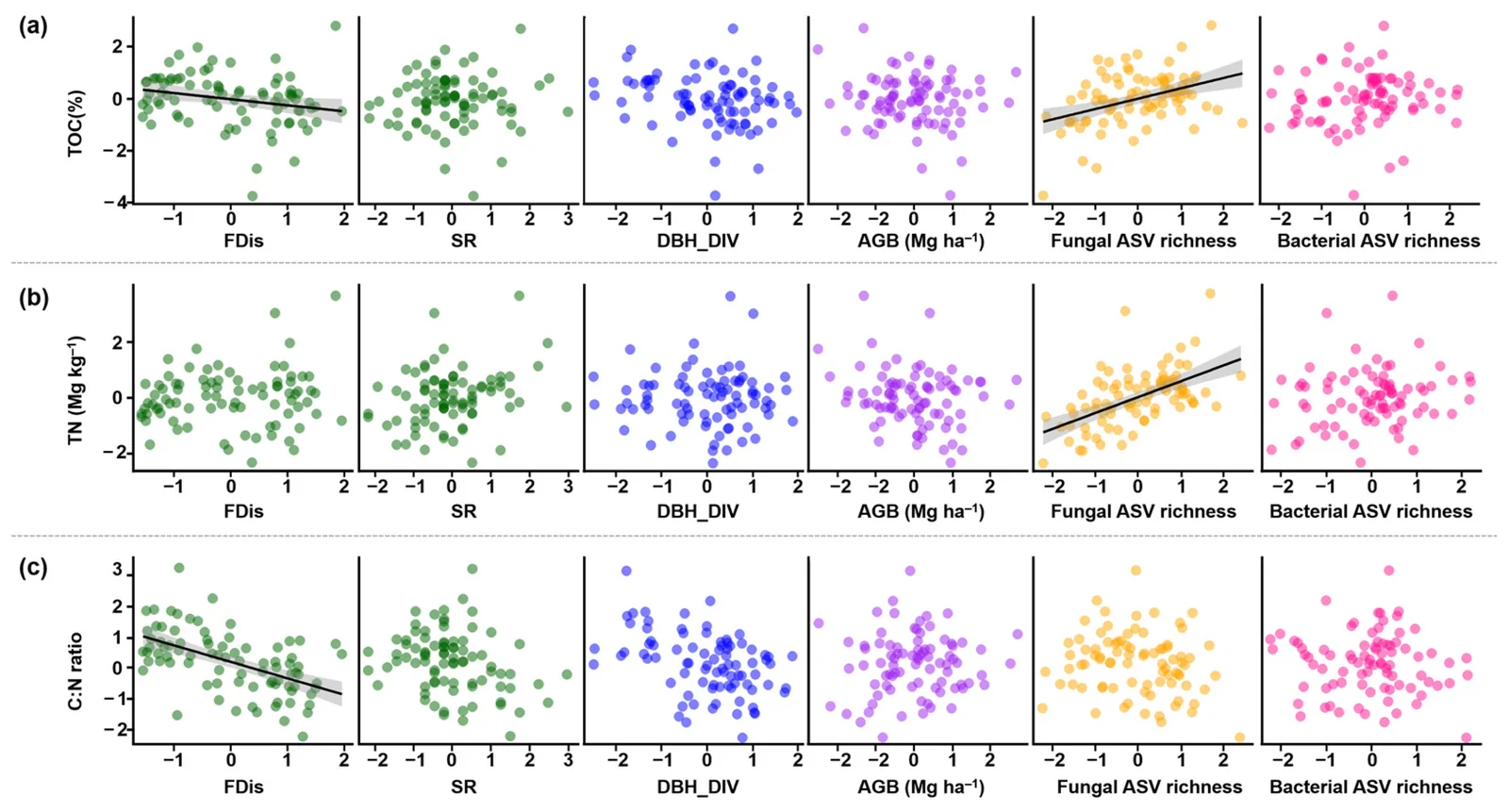
Uncorrected bivariate relationships of resource quality, resource quantity, and soil microbial communities with (**a**) total organic carbon (TOC), (**b**) total nitrogen (TN), and (**c**) the C:N ratio. These plots do not account for forest type, other covariates, or unmeasured environmental variables, and are shown for illustrative purposes only. Significant regressions are shown with fitted lines (*p* < 0.05), with shaded areas representing 95% confidence intervals. Abbreviations for the variables are provided in Figure 1.

As an uncorrected visual illustration, Figure 5 broadly reflects the directionality of the model-based associations reported in Figure 3 and Figure 4. However, it does not account for forest type or other covariates and should therefore not be interpreted as independent statistical confirmation of those results. Marginal (R^2^_m_) and conditional (R^2^_c_) coefficients of determination were highly similar across all models, indicating that forest type contributed little additional explanatory power.

## 4. Discussion

Our findings indicate that resource quality, rather than resource quantity, was more strongly associated with SOM dynamics, with an indirect statistical pathway involving fungal ASV richness playing a central role in the statistical association linking aboveground biodiversity to belowground carbon and nitrogen accumulation.

### 4.1. Resource Quality Rather than Resource Quantity Is Associated with Soil Organic Matter Dynamics

The negative relationship observed between FDis and TOC and the C:N ratio could plausibly be explained not only by the effect of functional diversity per se, but also by the influence of dominant tree species, although this remains a post hoc interpretation that our data cannot directly test. In communities with a low FDis, a small number of species tend to dominate, which is expected to be associated with the continuous input of litter with similar chemical characteristics [49]. Such homogeneous litter inputs could, in principle, be associated with the accumulation and stabilization of soil carbon [50]. In particular, if dominant species produce large amounts of litter containing high concentrations of recalcitrant compounds such as lignin and tannins, decomposition rates would be expected to be lower, which, in turn, would be consistent with a longer residence time of organic carbon in the soil and, accordingly, with greater TOC accumulation [51,52]. These litter characteristics could similarly be expected to be associated with higher soil C:N ratios, consistent with slower nitrogen mineralization and nutrient cycling processes. Indeed, litter chemical traits, including lignin, tannin, cutin, and suberin contents, have been widely recognized as important factors associated with long-term soil carbon storage [53,54]. Furthermore, considering the stand composition of the study area, these patterns may be associated with the dominance of coniferous species such as *Pinus densiflora*, *Pinus koraiensis*, and *Larix kaempferi*, and oak forests dominated by *Quercus mongolica*. These species are generally known to produce litter with relatively high concentrations of recalcitrant compounds, including lignin, tannins, waxes, and cutin [55,56]. On this basis, we hypothesize that their dominance may have been associated with the increases in both TOC and the soil C:N ratio observed in this study, though this could not be confirmed in the present study. These results are also consistent with previous studies suggesting that litter quality may play a more important role than litter quantity in the formation and accumulation of SOM [57,58]. Together, these findings suggest a plausible, though untested, explanation: that the increases in TOC and the C:N ratio observed in this study may reflect not only the effects of functional diversity itself, but also the chemical characteristics of litter supplied by the species that dominated under low FDis conditions [59]. However, because litter chemical composition, litterfall, dominant species litter traits, and SOM fractions were not directly measured in this study, this interpretation remains a hypothesis rather than a demonstrated mechanism. This interpretation is also consistent with the mass ratio hypothesis, which proposes that ecosystem functioning can be strongly associated with the functional traits of dominant species rather than by the overall diversity of the community [19,49]. It is important to note, however, that FDis reflects trait dispersion rather than the dominant functional identity of the community; a low-FDis community could, in principle, be dominated by species with either recalcitrant or labile litter traits. Because community-weighted mean (CWM) trait values were not evaluated in this study, the proposed mass-ratio-based mechanism cannot be directly confirmed with our current analysis; the dominant-species-related pathway should therefore be regarded as a hypothesis to be tested explicitly in future work.

AGB, representing the quantitative dimension of resource availability, was positively associated with DBH diversity but negatively associated with FDis. This pattern suggests that greater biomass production may occur in communities characterized by high structural complexity despite relatively low functional dispersion, and is broadly consistent with the niche complementarity hypothesis, which predicts enhanced productivity through more efficient light interception, spatial occupation, and resource acquisition in structurally complex communities [60,61]. However, increased AGB was not significantly related to SOM accumulation. This suggests that greater aboveground carbon storage does not necessarily translate into long-term carbon sequestration in belowground soil pools [62,63]. Rather, soil carbon accumulation appears to be more closely associated with resource quality than with resource quantity, suggesting that the chemical characteristics of litter input may play a more important role than the amount of AGB produced in determining carbon persistence in soils.

The results of this study suggest that forest soil carbon storage does not appear to be explained solely by biomass production, and that species composition and the traits of dominant species may also warrant attention in forest management. However, because a negative statistical association between FDis and TOC does not, by itself, establish which specific traits or species drive this pattern, our findings should not be interpreted as a general recommendation to either increase or decrease functional diversity for carbon management purposes. Rather, a more appropriate conclusion is that species composition, the functional traits of dominant species, and associated fungal communities may be important, and potentially interacting, factors influencing SOM dynamics that warrant further empirical investigation before being translated into specific management guidance. Identifying which species or traits are actually associated with greater carbon stabilization in this system would require direct measurement of litter chemistry and community-weighted trait values, which are beyond the scope of the present study.

### 4.2. Species Richness Is Linked to Total Soil Organic Carbon and Total Nitrogen Accumulation via an Indirect Fungal Pathway

Our results indicate a statistical indirect pathway in which fungal ASV richness is statistically linked to aboveground species diversity (i.e., species richness) and to belowground carbon and nitrogen accumulation. It should be noted that fungal community characteristics in this study were represented solely by ASV richness, a measure of taxonomic diversity; fungal biomass, activity, and necromass were not directly measured, and the following mechanistic interpretations should therefore be regarded as possible explanations rather than processes demonstrated by our data. In forest ecosystems, increasing species richness could plausibly be associated with greater diversity of plant-derived organic substrates entering the soil through litter input, fine-root turnover, and root exudation, which, in turn, might be linked to greater resource heterogeneity and a broader range of fungal taxa [64]. Such conditions could, in principle, be associated with increased fungal activity and with the transformation of plant-derived organic matter into microbially derived organic matter, which has been widely recognized as a major contributor to the formation and stabilization of SOM [65,66].

The positive association between fungal ASV richness and TOC and TN is consistent with several complementary mechanisms. First, fungi possess extracellular enzymes (e.g., lignin peroxidase and laccase) capable of degrading recalcitrant organic compounds such as lignin, which are largely inaccessible to bacteria [67]. Through this process, fungi decompose plant-derived organic inputs and convert them into microbial biomass, which can subsequently accumulate as microbial necromass following cell death [68]. Fungal necromass is widely recognized as a major precursor of persistent soil organic matter, owing to the presence of relatively recalcitrant cell-wall constituents, such as chitin and melanin, which are resistant to microbial decomposition [69]. As a result, greater fungal biomass may be associated with the long-term sequestration and accumulation of both carbon and nitrogen in forest soils [70]. Second, although not directly measured in this study, fungal hyphae have been reported to be physically associated with soil aggregation [71]. Extensive hyphal networks bind mineral particles and organic matter together, which is consistent with the formation of stable soil aggregates that protect organic carbon and nitrogen from microbial decomposition and losses through leaching [72]. Through this proposed mechanism, fungal communities could plausibly function as a biological filter associated with the retention of nutrients and organic matter in forest soils [73]. Finally, symbiotic fungi, particularly mycorrhizal fungi, have been reported in the literature to be associated with nitrogen retention through nutrient exchange between plants and the soil [74]. A comparable process, if present in our study system, could plausibly be linked to reduced nitrogen loss and, correspondingly, to the higher TN concentrations observed in stands with high species richness [75].

In contrast, bacterial ASV richness showed no significant relationship with TOC, TN, or the C:N ratio despite being statistically linked by SR. This could be consistent with bacteria being primarily involved in rapid nutrient-cycling processes, such as organic matter decomposition, mineralization, and nitrification, rather than in the long-term storage of nitrogen [76]. In addition, bacterial-derived necromass is generally characterized by faster turnover and lower persistence in soils than fungal necromass, which could plausibly limit its contribution to long-term nitrogen accumulation [77]. Therefore, the SOM dynamics observed in this study are more closely associated with fungal statistical pathways identified in the pSEM than with bacterial processes. As noted above, however, the biological mechanisms underlying this pathway, including fungal biomass, activity, and necromass formation, were not directly measured in this study and remain to be tested empirically.

Overall, this study indicates that resource quality was more strongly associated with SOM dynamics than resource quantity, with an indirect statistical pathway involving fungal richness playing a central role in temperate forest soils. By integrating aboveground vegetation characteristics with belowground microbial processes, our findings provide new insights into the statistical pathways associated with biodiversity and soil carbon and nitrogen storage. These results highlight the importance of considering not only biomass production but also species composition and functional characteristics in forest management strategies aimed at enhancing long-term soil carbon sequestration. Ultimately, our study contributes to the development of more effective biodiversity conservation and sustainable forest soil management strategies under ongoing climate change.

### 4.3. Limitations and Future Research Directions

This study has several limitations that should be considered when interpreting the observed relationships. First, resource quality was represented by proxy variables, including species richness and functional dispersion, rather than by direct measurements of litter chemical traits such as lignin, cellulose, tannins, or nutrient concentrations. Likewise, resource quantity was inferred from aboveground biomass and DBH diversity rather than from direct measurements of litterfall, fine root production, or other organic matter inputs to the soil. Therefore, the terms “resource quality” and “resource quantity” in this study should be interpreted as conceptual proxies rather than direct measurements of organic matter quality and flux. Second, soil microbial communities were characterized using fungal and bacterial ASV richness. These sequence-derived metrics represent detected taxonomic richness but do not directly quantify microbial abundance, biomass, metabolic activity, functional groups, or functional traits. Accordingly, the positive associations of fungal ASV richness with TOC and TN should not be interpreted as evidence of greater fungal biomass, activity, or necromass formation. Third, several potentially important abiotic and historical variables, including soil pH, texture, moisture, elevation, slope, aspect, microclimate, stand age, land-use history, or mycorrhizal association type, were not consistently available. These variables may simultaneously influence vegetation characteristics, soil microbial communities, and SOM and could therefore act as unmeasured common drivers of the observed relationships. Accordingly, we cannot rule out the possibility that some of the indirect pathways identified in the pSEM partly reflect environmental covariation rather than independent effects of the vegetation resource proxies themselves. Fourth, the pSEM analyses were based on observational and cross-sectional data. Although the model structure was developed from a priori conceptual framework, the resulting pathways represent conditional statistical associations rather than experimentally demonstrated causal relationships. In addition, model simplification based on the statistical support of individual paths may introduce some uncertainty in model selection. Therefore, the direct and indirect pathways identified in this study should be interpreted cautiously and require further validation using longitudinal observations, independent datasets, or experimental manipulations. Fifth, SOM dynamics were evaluated using TOC, TN, and the C:N ratio, which represent important but limited dimensions of soil organic matter status and do not fully capture SOM stabilization, fractionation, or turnover processes.

Future studies should incorporate direct measurements of litter chemical properties and litter decomposition characteristics to more accurately quantify resource quality. Likewise, direct measurements of litterfall, fine-root production and turnover, and other belowground carbon inputs would provide more robust estimates of resource quantity. Integrating measurements of microbial biomass, functional groups, and extracellular enzyme activities with metagenomic or metatranscriptomic approaches would further clarify the microbial pathways associated with SOM formation and stabilization. In addition, measurements of soil physical properties, including aggregate stability and bulk density, would help evaluate the potential contribution of physical protection mechanisms to SOM stabilization. Expanding SOM assessments to include particulate organic matter, mineral-associated organic matter fractions, dissolved organic carbon, microbial biomass carbon, and direct measures of carbon turnover would also provide a more comprehensive understanding of SOM formation, persistence, and stabilization. Ultimately, long-term observations and manipulative experiments integrating vegetation inputs, microbial processes, soil physicochemical conditions, and distinct SOM pools will be needed to determine whether the statistical relationships identified in this study reflect underlying causal mechanisms.

## 5. Conclusions

This study examined, within the 84 plots sampled across six forest types in Mt. Gariwang, South Korea, whether SOM dynamics were more strongly associated with resource quality, indirectly represented by species richness and functional dispersion, or resource quantity, represented by AGB and DBH diversity, while also evaluating the statistical indirect pathways involving soil microbial communities, including fungi and bacteria. Within the observed range of variation in this study system, resource quality showed stronger associations with SOM dynamics than resource quantity. FDis was directly associated with TOC and the C:N ratio, a pattern potentially consistent with the accumulation of recalcitrant litter from dominant species under low functional diversity. However, the functional identity of these dominant species was not directly assessed, whereas AGB showed no significant relationships with SOM indicators. In addition, SR was associated with fungal ASV richness, which, in turn, showed a positive statistical association with both TOC and TN, which is consistent with an indirect pathway in the pSEM linking aboveground biodiversity to belowground carbon and nitrogen accumulation; it should be noted that this “mediation” is statistical in nature, reflecting the model structure, and does not itself demonstrate the underlying biological mechanism. Our findings suggest that, in this study system, SOM dynamics were associated not only with the amount of plant-derived organic input but also with proxies for their quality and with fungal ASV richness relationships that require confirmation through direct process measurements (e.g., litter chemistry, community-weighted trait values, fungal biomass and activity) and long-term studies before their underlying mechanisms can be considered established. Because FDis showed a negative, rather than positive, association with TOC and the C:N ratio in this study, our results should not be taken to suggest that increasing functional diversity per se would enhance soil carbon storage. Rather, a more cautious conclusion is that species composition, the functional identity of dominant species, and fungal ASV richness may be important correlates of SOM dynamics that warrant further investigation, rather than factors whose management implications can yet be specified. Any management inferences drawn from these findings should therefore be considered provisional and specific to forest systems comparable to the one studied here. Further studies integrating direct measurements of litter chemistry, plant functional identity, microbial functional traits, and long-term observations are needed to determine whether the associations identified here reflect causal mechanisms, and to support more effective biodiversity conservation and sustainable forest management under changing environmental conditions.

## Data Availability

The original contributions presented in the study are available in Supplementary Material S1. Further inquiries can be directed to the corresponding author, Chang-Bae Lee.

## Supplementary Materials

The following supporting information can be downloaded at: https://www.mdpi.com/article/10.3390/biology15181568/s1, Figure S1: Box plots for the comparison of resource quality, resource quantity, soil microbiota, and soil organic matter dynamics indices, including (a) FDis, (b) SR, (c) DBH_DIV, (d) AGB, (e) Fungal ASV richness, (f) Bacterial ASV richness, (g) TOC, (h) TN, (i) and the C:N ratio among six forest types (i.e., *Quercus mongolica, Larix Kaempferi, Pinus densiflora, Pinus Koraiensis*, *Other Quercus*, and *Other Broadleaved*) in Gariwang Mountain in South Korea. Abbreviations for all variables are provided in Table S3; Figure S2: Pearson’s correlation coefficients between resource quality, resource quantity, soil microbiota, and (a) TOC, (b) TN, and (c) the C:N ratio in the study plots (*n* = 84). Abbreviations of the variables are provided in Table S3; Table S1: Functional trait data and allometric equations for the estimation of the aboveground biomass (AGB) of the woody plant species recorded in this study. Scientific names follow The WFO Plant List (www.wfoplantlist.org). Functional traits and AGB equations were obtained from the listed literature and open-access online databases. Abbreviations: SLA, specific leaf area; WD, wood density; MH, maximum height; N, leaf nitrogen content; C, leaf carbon content. * Note: Table S1 was prepared in a different file (i.e., an Excel file); Table S2: Fusion primer sequences used for PCR amplification of fungi and bacteria communities. Fusion primers were used for PCR amplification targeting the ITS2 region for fungi and the V3–V4 regions of the 16S rRNA gene for bacteria. The fusion primers were designed to include Illumina adapter sequences, index regions, and target-specific primer sequences. Each primer consisted of the P5 (forward) or P7 (reverse) binding site, i5 or i7 index, Nextera consensus sequence, sequencing adapter, and the target-specific region. Detailed primer sequences are provided in this table; Table S3: Summary of indices for resource quality, resource quantity, soil microbiota, and soil organic matter dynamics across total stand types in Gariwang Mountain in South Korea. Abbreviations: Min, minimum; Max, maximum; SE, standard error; FDis, functional dispersion; SR, species richness; DBH, the diameter of breast height; DIV, diversity; AGB, aboveground biomass; TOC, total soil organic carbon; TN, total nitrogen content; C:N ratio, Total organic carbon-to-total nitrogen content ratio; Table S4: Summary of the generalized least−squares (GLS) models to test spatial autocorrelation for resource quality, resource quantity, and soil microbiota with TOC, TN, and the C:N ratio. The non-spatial and spatial models were implemented with and without geographic coordinates (i.e., latitude and longitude) of plots, respectively. Abbreviations for all variables are provided in Table S3; Table S5. Model-averaged parameter estimates and relative importance of resource quality, resource quantity, and soil microbiota predictors for TOC, TN, and the C:N ratio. Abbreviations: CI, confidence interval; AICc, Corrected Akaike Information Criterion. Other abbreviations are provided in Table S3.
