# Supplementary material for: Resource Quality and Fungal Richness Are Associated with Soil Organic Matter Dynamics in a Temperate Forest in South Korea
Source: Biology (Basel). 2026 Sep 8;15(18):1568. doi: 10.3390/biology15181568 (PMC13603730; doi:10.3390/biology15181568)

Methods

Soil microbial sequence processing

Raw paired-end reads from fungal and bacterial amplicon sequencing were processed using a DADA2-based bioinformatics pipeline. Adapter and primer sequences were removed using Cutadapt v3.2. Sequence quality filtering and error-model-based denoising were subsequently performed using DADA2 v1.18.0, with reads having expected errors >2 excluded. Forward and reverse reads were merged after denoising, and chimeric sequences were identified and removed using the consensus method implemented in the removeBimeraDenovo function of DADA2.

For the bacterial 16S dataset, forward and reverse reads were trimmed to 250 and 200 bp, respectively, and ASVs shorter than 350 bp were additionally removed using R v4.0.3. No corresponding ASV-length filtering was applied to the fungal ITS dataset. No additional abundance- or prevalence-based filtering of rare ASVs was conducted after these quality-control procedures.

Sequencing depth was standardized within each sequencing batch by rarefaction to the minimum retained read count using QIIME v1.9.0. Fungal ASVs were taxonomically assigned using UCLUST against the UNITE Fungi2 99% Clustering database (v8.2, 20 February 2020). An additional taxonomic assignment against the UNITE+INSD All Eukaryotes database (v8.2, 4 February 2020) was generated as part of the fungal sequencing pipeline. Bacterial ASVs were taxonomically assigned using BLAST against the NCBI 16S reference database (NCBI_16S_20220526; release 26 May 2022).

Fungal and bacterial ASV richness was calculated as the number of detected ASVs in each plot after sequencing-depth standardization and was used as the microbial diversity metric in the subsequent statistical analyses.

Reference

39. Martin, M. Cutadapt removes adapter sequences from high-throughput sequencing reads. *EMBnet. j.* **2011**, *17*, 10–12.

40. Callahan, B.J.; McMurdie, P.J.; Rosen, M.J.; Han, A.W.; Johnson, A.J.A.; Holmes, S.P. DADA2: high-resolution sample inference from Illumina amplicon data. *Nat. Methods* **2016**, *13*, 581–583.

41. Caporaso, J.G.; Kuczynski, J.; Stombaugh, J.; Bittinger, K.; Bushman, F.D.; Costello, E.K.; Knight, R. QIIME allows analysis of high-throughput community sequencing data. *Nat. Methods* **2010**, *7*, 335–336.

List of Supplementary Tables & Figures

Table S1. Functional trait data and allometric equations for the estimation of the aboveground biomass (AGB) of the woody plant species recorded in this study. Scientific names follow The WFO Plant List (www.wfoplantlist.org). Functional traits and AGB equations were obtained from the listed literature and open access online database. Abbreviations: SLA, specific leaf area; WD, wood density; MH, maximun height; N, leaf nitrogen content; C, leaf carbon content.

* *Note: Table S1 was prepared in a different file (i.e., excel file).*

Table S2. Fusion primer sequences used for PCR amplification of fungi and bacteria communities. Fusion primers were used for PCR amplification targeting the ITS2 region for fungi and the V3–V4 regions of the 16S rRNA gene for bacteria. The fusion primers were designed to include Illumina adapter sequences, index regions, and target-specific primer sequences. Each primer consisted of the P5 (forward) or P7 (reverse) binding site, i5 or i7 index, Nextera consensus sequence, sequencing adapter, and the target-specific region. Detailed primer sequences are provided in this table.

Table S3. Summary of indices for resource quality, resource quantity, soil microbiota and soil organic matter dynamics across total stand types in Gariwang Mountain in South Korea. Abbreviations: Min, minimum; Max, maximum; SE, standard error; FDis, functional dispersion; SR, species richness; DBH, the diameter of breast height; DIV, diversity; AGB, aboveground biomass; TOC, total soil organic carbon; TN, total nitrogen content; C:N ratio, Total organic carbon-to-total nitrogen content ratio.

Table S4. Summary of the generalized least−squares (GLS) models to test spatial autocorrelation for resource quality, resource quantity and soil microbiota with TOC, TN and C:N ratio. The non−spatial and spatial models were implemented with and without geographic coordinates (i.e., latitude and longitude) of plots, respectively. Abbreviations for all variables are provided in Table S3.

Table S5. Model-averaged parameter estimates and relative importance of resource quality, resource quantity, and soil microbiota predictors for TOC, TN, and the C:N ratio. Abbreviations: CI, confidence interval; AICc, Corrected Akaike Information Criterion. Other abbreviations are provided in Table S3.

Figure S1. Box plots for the comparison of resource quality, resource quantity, soil microbiota and soil organic matter dynamics indices including (a) FDis, (b) SR, (c) DBH_DIV, (d) AGB, (e) Fungi, (f) Bacteria, (g) TOC, (h) TN, (i) C:N ratio among six forest types (i.e., *Quercus mongolica, Larix Kaempferi, Pinus densiflora, Pinus Koraiensis*, *Other Quercus* and *Other Broadleaved*) in Gariwang Mountain in South Korea. Abbreviations for all variables are provided in Table S3.

Figure S2. Pearson's correlation coefficients between resource quality, resource quantity, soil microbiota, and (a) TOC, (b) TN, and (c) C:N ratio in the study plots (n = 84). Abbreviations of the variables are provided in Table S3.

Table S2. Fusion primer sequences used for PCR amplification of fungi and bacteria communities. Fusion primers were used for PCR amplification targeting the ITS2 region for fungi and the V3–V4 regions of the 16S rRNA gene for bacteria. The fusion primers were designed to include Illumina adapter sequences, index regions, and target-specific primer sequences. Each primer consisted of the P5 (forward) or P7 (reverse) binding site, i5 or i7 index, Nextera consensus sequence, sequencing adapter, and the target-specific region. Detailed primer sequences are provided in this table.

Target	Primer name	Direction	Sequence (5'-3')	Target region
Fungi	ITS3	Forward	AATGATACGGCGACCACCGAGATCTACACXXXXXXXXTCGTCGGCAGCGTC-AGATGTGTATAAGAGACAGGCATCGATGAAGAACGCAGC	ITS2
	ITS4	Reverse	CAAGCAGAAGACGGCATACGAGATXXXXXXXXGTCTCGTGGGCTCGG-AGATGTGTATAAGAGACAGTCCTCCGCTTATTGATATGC	
Bacteria	341F	Forward	AATGATACGGCGACCACCGAGATCTACACXXXXXXXXTCGTCGGCAGCGTCAGATGTGTATAAGAGACAGCCTACGGGNGGCWGCAG	V3-V4
	805R	Reverse	CAAGCAGAAGACGGCATACGAGATXXXXXXXXGTCTCGTGGGCTCGGAGATGTGTATAAGAGACAGGACTACHVGGGTATCTAATCC	

Table S3. Summary of indices for resource quality, resource quantity, soil microbiota and soil organic matter dynamics across total stand types in Gariwang Mountain in South Korea. Abbreviations: Min, minimum; Max, maximum; SE, standard error; FDis, functional dispersion; SR, species richness; DBH, the diameter of breast height; DIV, diversity; AGB, aboveground biomass; TOC, total soil organic carbon; TN, total nitrogen content; C:N ratio, Total organic carbon-to-total nitrogen content ratio.

Variables	Unit	Total stand (n=84)			
		Min	Max	Mean	SE
Resource quality					
FDis	unitless	0	1.76	0.78	0.05
SR	unitless	1	22	9.81	0.45
Resource quantity					
DBH DIV	unitless	1.54	3.29	2.53	0.04
AGB	mg ha^{-1}	4147.21	18703.79	8992.64	295.1
Soil microbiota					
Fungal	ASV richness	325	1676	759.69	28.88
Bacterial	ASV richness	805	1905	1268.93	26.81
Soil organic matter dynamics					
TOC	%	2.75	17.67	6.75	0.23
TN	mg kg^{-1}	0.28	1.25	0.53	0.02
C:N ratio	unitless	7.98	20.48	12.93	0.24

Table S4. Summary of the generalized least−squares (GLS) models to test spatial autocorrelation for resource quality, resource quantity and soil microbiota with TOC, TN and the C:N ratio. The non−spatial and spatial models were implemented with and without geographic coordinates (i.e., latitude and longitude) of plots, respectively. Abbreviations for all variables are provided in Table S3.

GLS model	Model	Coefficient	t−value	P−value	AIC	R^2_{pseudo}
Total soil organic carbon (TOC)						
TOC ~ FDis	Non−spatial	− 0.235	−2.188	0.032	243.9	0.055
	Spatial	− 0.108	− 0.833	0.407	239.81	0.009
TOC ~ SR	Non−spatial	0.031	0.283	0.778	248.47	0.001
	Spatial	0.067	0.577	0.565	240.31	0.002
TOC ~ DBH_DIV	Non−spatial	− 0.247	− 2.305	0.024	243.4	0.061
	Spatial	− 0.11	− 0.865	0.39	239.75	0.001
TOC ~ AGB	Non−spatial	− 0.129	− 1.172	0.244	247.19	0.017
	Spatial	− 0.085	− 0.792	0.431	240.14	0.008
TOC ~ Fungal ASV richness	Non−spatial	0.399	3.934	< 0.001	234.37	0.159
	Spatial	0.392	3.68	< 0.001	229.15	0.125
TOC ~Bacterial ASV richness	Non−spatial	0.084	0.763	0.448	247.97	0.007
	Spatial	0.039	0.344	0.732	24.54	0.001
Total nitrogen content (TN)						
TN ~ FDis	Non−spatial	0.124	1.134	0.26	247.28	0.016
	Spatial	0.218	1.674	0.098	235.04	0.033

TN ~ SR	Non−spatial	0.259	2.428	0.018	242.86	0.067
	Spatial	0.168	1.475	0.144	236.06	0.025
TN ~ DBH_DIV	Non−spatial	0.001	0.013	0.99	248.55	< 0.001
	Spatial	0.032	0.25	0.804	237.79	< 0.001
TN ~ AGB	Non−spatial	− 0.21	− 1.944	0.055	244.86	0.044
	Spatial	− 0.185	− 1.775	0.08	235.26	0.037
TN ~ Fungal ASV richness	Non−spatial	0.557	6.069	< 0.001	218.13	0.31
	Spatial	0.501	5.052	0	219.7	0.207
TN ~ Bacterial ASV richness	Non−spatial	0.151	1.387	0.169	246.65	0.023
	Spatial	0.019	0.172	0.863	238.12	0.001
Total organic carbon-to-total nitrogen content ratio (C:N ratio)						
C:N ratio ~ FDis	Non−spatial	− 0.537	− 5.755	0	220.73	0.288
	Spatial	−0.536	− 5.732	0	224.55	0.099
C:N ratio ~ SR	Non−spatial	− 0.269	− 2.532	0.013	242.38	0.073
	Spatial	− 0.145	− 1.447	0.152	226.61	0.004
C:N ratio ~ DBH_DIV	Non−spatial	− 0.426	− 4.261	< 0.001	232.15	0.181
	Spatial	− 0.422	− 4.212	< 0.001	236.07	− 0.037
C:N ratio ~ AGB	Non−spatial	0.069	0.627	0.533	248.16	0.005
	Spatial	0.095	0.963	0.338	227.73	0.011
C:N ratio ~ Fungal ASV richness	Non−spatial	− 0.161	− 1.476	0.143	246.4	0.026
	Spatial	− 0.034	− 0.335	0.739	228.47	0.001

C:N ratio ~ Bacterial ASV richness	Non−spatial	− 0.082	− 0.743	0.46	248	0.007
	Spatial	0.097	0.91	0.366	227.67	− 0.003

Table S5. Model-averaged parameter estimates and relative importance of resource quality, resource quantity, and soil microbiota predictors for TOC, TN, and the C:N ratio. Abbreviations: CI, confidence interval; AICc, Corrected Akaike Information Criterion. Other abbreviations are provided in Table S3.

- **(a) Multi-model inference results for total organic carbon (TOC): Model-averaged parameter estimates (conditional average, 32 candidate models)**

Predictor	Estimate (β)	Std. Error	Adjusted SE	Z value	p-value	95% CI	Relative importance (%)
Fungal ASV richness	0.448	0.119	0.12	3.75	<0.001 ***	0.212, 0.684	42
Bacterial ASV richness	−0.132	0.113	0.115	1.155	0.248	−0.357, 0.092	12.4
FDis	−0.262	0.121	0.123	2.134	0.033 *	−0.502, −0.021	24.5
SR	−0.073	0.132	0.134	0.547	0.584	−0.335, 0.189	6.9
DBH diversity	−0.150	0.127	0.129	1.158	0.247	−0.403, 0.104	14
AGB	−0.002	0.107	0.108	0.019	0.985	−0.214, 0.210	0.2

*Best-supported candidate model (FDis + Fungal ASV richness): AICc = 223.71, weight = 0.16.

- **(b) Multi-model inference results for total nitrogen (TN): Model-averaged parameter estimates (conditional average, 64 candidate models)**

Predictor	Estimate (β)	Std. Error	Adjusted SE	Z value	p-value	95% CI	Relative importance (%)
Fungal ASV richness	0.567	0.104	0.106	5.357	<0.001 ***	0.360, 0.775	67.7
Bacterial ASV richness	−0.140	0.106	0.107	1.305	0.192	−0.350, 0.070	16.7
FDis	0.030	0.106	0.108	0.283	0.777	−0.180, 0.241	3.6
SR	0.007	0.111	0.112	0.061	0.952	−0.214, 0.227	0.8
DBH diversity	−0.016	0.103	0.105	0.153	0.878	−0.222, 0.190	1.9
AGB	−0.078	0.097	0.098	0.791	0.429	−0.270, 0.115	9.3

*Best-supported candidate model (Fungal ASV richness): AICc = 214.72, weight = 0.18.

– **(c) Multi-model inference results for C:N ratio: Model-averaged parameter estimates (conditional average, 64 candidate models)**

Predictor	Estimate (β)	Std. Error	Adjusted SE	Z value	p-value	95% CI	Relative importance (%)
Fungal ASV richness	−0.061	0.104	0.105	0.579	0.563	−0.268, 0.146	6.7
Bacterial ASV richness	−0.047	0.1	0.102	0.458	0.647	−0.246, 0.153	5.1
FDis	−0.446	0.126	0.128	3.478	<0.001 ***	−0.698, −0.195	49.1
SR	−0.05	0.116	0.118	0.421	0.674	−0.281, 0.182	5.5
DBH diversity	−0.206	0.125	0.126	1.626	0.104	−0.453, 0.042	22.6
AGB	0.099	0.106	0.107	0.924	0.356	−0.111, 0.310	10.9

*Best-supported candidate model (DBH diversity+FDis): AICc = 217.44, weight = 0.13.

Figure S1. Box plots for the comparison of resource quality, resource quantity, soil microbiota and soil organic matter dynamics indices including (a) FDis, (b) SR, (c) DBH_DIV, (d) AGB, (e) Fungal ASV richness, (f) Bacteria ASV richness, (g) TOC, (h) TN, (i) and the C:N ratio among six forest types (i.e., *Quercus mongolica, Larix Kaempferi, Pinus densiflora, Pinus Koraiensis*, *Other Quercus* and *Other Broadleaved*) in Gariwang Mountain in South Korea. Abbreviations for all variables are provided in Table S3.

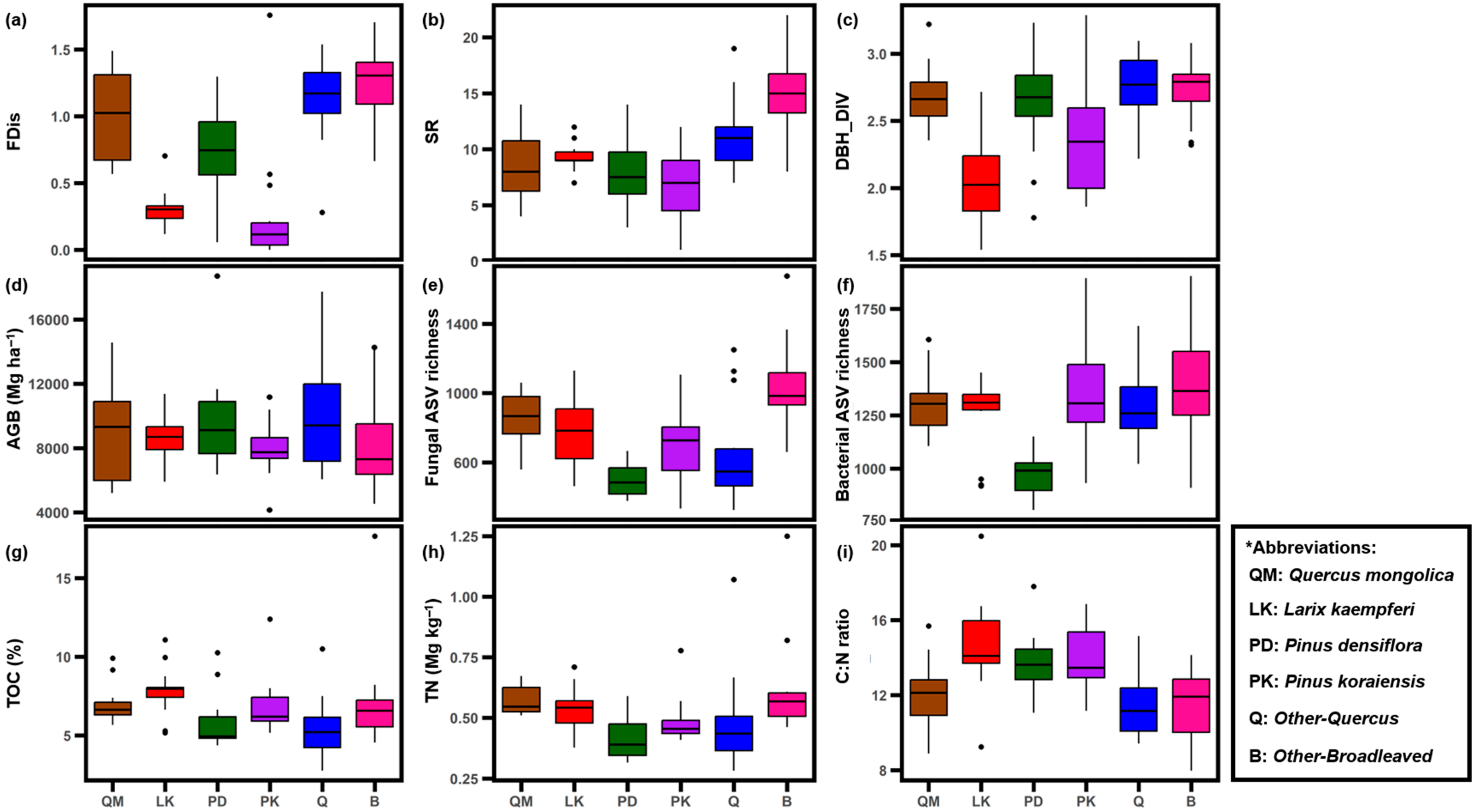

Figure S2. Pearson's correlation coefficients between resource quality, resource quantity, soil microbiota, and (a) TOC, (b) TN, and (c) the C:N ratio in the study plots (n = 84). Abbreviations of the variables are provided in Table S3.

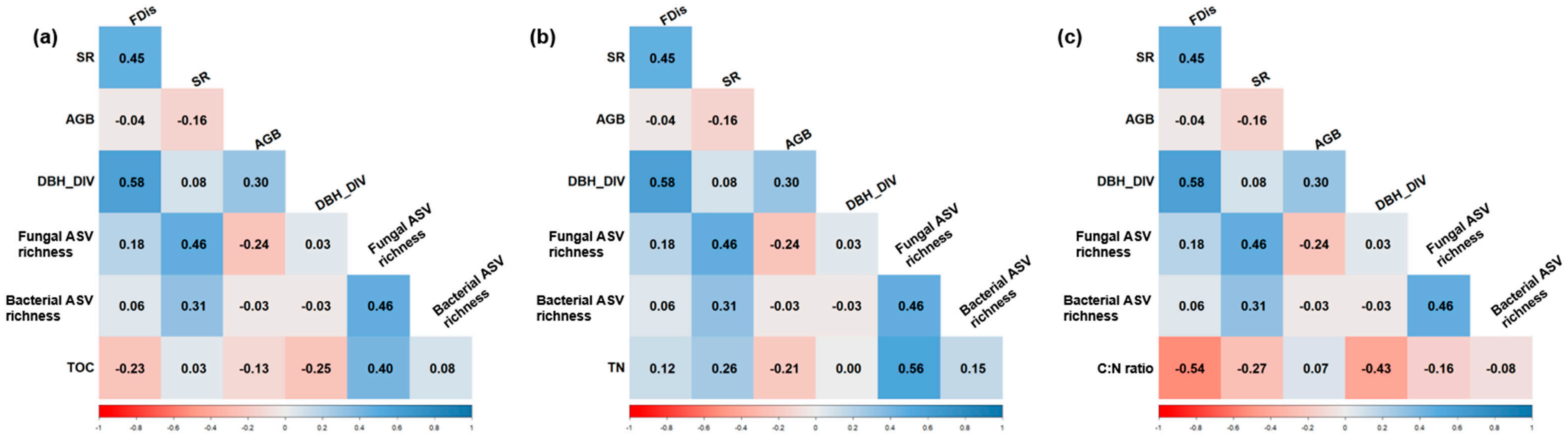

Supplement: Supplementary file 1 [file biology-15-01568-s001.zip › Supplementary Material S2.pdf]
